# A DNAmCULTURE Epigenetic Fingerprint Recapitulates Physiological Aging

**DOI:** 10.1093/geroni/igab046.018

**Published:** 2021-12-17

**Authors:** Christopher Minteer, Marco Morselli, Margarita Meer, Jian Cao, Sabine Lang, Matteo Pellegrini, Qin Yan, Morgan Levine

**Affiliations:** 1 Yale University, Yale University, Connecticut, United States; 2 UCLA, Los Angeles, California, United States; 3 Yale School of Medicine, New Haven, Connecticut, United States; 4 Rutgers University, Rutgers University, New Jersey, United States; 5 Yale University, New Haven, Connecticut, United States; 6 UCLA, UCLA, California, United States

## Abstract

Aging elicits dramatic changes to DNA methylation (DNAm), however the causes and consequences of such alterations to the epigenome remain unclear. The utility of biomarkers of aging based on DNAm patterns would be greatly enhanced if in vitro models existed that recapitulated physiological phenotypes such that modulation could garnish mechanistic insights. Using DNAm from serially passaged mouse embryonic fibroblasts, we developed a marker of culture aging and asked if culture phenotypes, like exhaustive replication, are epigenetically analogous to physiological aging. Our measure, termed DNAmCULTURE, accurately estimated passage number and was shown to strongly increase with age when examined in multiple tissues. Furthermore, we observed epigenetic alterations indicative of early cultured cells in animals undergoing caloric restriction and in lung and kidney fibroblasts re-programmed to iPSCs. This study identifies culture-derived alterations to the methylome as physiologically relevant and implicates culture aging as an important feature in known epigenetic aging phenomena.

